# Priming thermotolerance: unlocking heat resilience for climate-smart crops

**DOI:** 10.1098/rstb.2024.0234

**Published:** 2025-05-29

**Authors:** Priyanka Chopra, Natalia Sapia, Omid Karami, Pawan Kumar, David Honys, Lucia Colombo, Marta Mendes, Moussa Benhamed, Vasileios Fotopoulos, Michal Lieberman-Lazarovich, Bernd Mueller-Roeber, Eirini Kaiserli, Said Hafidh, Sotirios Fragkostefanakis

**Affiliations:** ^1^ Institute of Biology Leiden, Leiden University, Leiden, The Netherlands; ^2^ Institute of Molecular Biosciences, Goethe-Universitat Frankfurt am Main, Frankfurt am Main, Germany; ^3^ Institute of Plant Sciences Paris-Saclay (IPS2), Universite Paris-Saclay, Gif-sur-Yvette, France; ^4^ Institute of Biochemistry and Biology, University of Potsdam, Potsdam, Germany; ^5^ Institute of Plant Sciences, Agricultural Research Organization, Rishon LeZion, Israel; ^6^ Institute of Experimental Botany, Czech Academy of Sciences, Prague, Czech Republic; ^7^ University of Milan, Milan, Italy; ^8^ Cyprus University of Technology, Limassol, Cyprus; ^9^ Molecular Cell and Systems Biology, University of Glasgow, Glasgow, UK

**Keywords:** crop resilience, thermotolerance, thermomemory, priming, heat stress, global warming

## Abstract

Rising temperatures and heat waves pose a substantial threat to crop productivity by disrupting essential physiological and reproductive processes. While plants have a genetically inherited capacity to acclimate to high temperatures, the thermotolerance capacity of many crops remains limited. This limitation leads to yield losses, which are further intensified by the increasing intensity of climate change. In this review, we explore how thermopriming enhances plant resilience by preparing plants for future heat stress (HS) events and summarize the mechanisms underlying the memory of HS (thermomemory) in different plant tissues and organs. We also discuss recent advances in priming agents, including chemical, microbial and physiological interventions, and their application strategies to extend thermotolerance beyond inherent genetic capacity. Additionally, this review examines how integrating priming strategies with genetic improvements, such as breeding and genome editing for thermotolerance traits, provides a holistic solution to mitigate the impact of climate change on agriculture. By combining these approaches, we propose a framework for developing climate-resilient crops and ensuring global food security in the face of escalating environmental challenges.

This article is part of the theme issue ‘Crops under stress: can we mitigate the impacts of climate change on agriculture and launch the ‘Resilience Revolution’?’.

## 1. Introduction

Plants experience heat stress (HS) when rising temperatures disrupt important physiological and biochemical processes. HS typically leads to growth inhibition and disruption of reproduction, resulting in reduced pollen viability, impaired fertilization and decreased seed or fruit set. Most crops are sensitive to high temperatures, and therefore long mild or short-term severe HS can cause major yield losses. Consequently, the higher intensity and frequency of heat waves, as one of the consequences of global warming, is a major threat for global food security [1,2].

Elevated temperatures trigger changes in plant metabolism and physiology, ensuring survival and facilitating the recovery from stress [3]. Survival depends largely on mechanisms that protect macromolecules and cellular structures, including DNA, proteins and membranes, from irreversible damage [4]. The recovery from HS depends on the ability of the plant to re-establish homeostasis and is characterized by the rebalancing of metabolic activities, removal of deleterious molecular species, restoration of photosynthetic efficiency, and the resumption of growth and developmental processes. Depending on the duration and severity of the HS the physiological acclimation strategies that aid survival and long-term acclimation include, among others, growth reduction for energy conservation, changes in organ morphology to improve heat dissipation capacity, increase in transpiration for cooling, and developmental alterations to accelerate or delay flowering and seed production as an avoidance strategy against heat [5,6].

Cells perceive temperature changes by an array of sensing mechanisms that are mainly based on changes in the redox state and the biophysical properties of RNA, proteins and membranes, which lead to the activation of HS response-relevant signalling and transcriptional networks [7–9]. These networks, while fundamentally conserved at their core, exhibit variations that depend on the specific organ, tissue and cell type and can also be influenced by various factors, including the type of stress imposed, the developmental stage and the physiological status of the plant.

Plants have a genetically determined intrinsic capacity to combat an acute HS incident, termed basal thermotolerance (BTT) [10]. BTT depends on the activity of a group of heat stress transcription factors (HSFs) responsible for triggering the expression of genes that are essential for the initial protection of cells from HS damages, such as heat shock proteins (HSPs) but also enzymes involved in scavenging reactive oxygen species (ROS) [11]. Among HSFs, HSFA1s function as master regulators of the HS response and thermotolerance [12,13]. Plants can acquire thermotolerance and survive stress impacts that are beyond their BTT limits, by a pre-exposure to a mild HS [14,15]. The presence of HSPs and HSFs in pre-acclimated plants offers an immediate protection against an otherwise lethal HS and stimulates gene expression and protein synthesis, which together result in acquired thermotolerance (ATT) [10]. ATT resembles the acclimation mechanisms that are activated during a hot day, when temperature gradually rises during the day and peaks at midday, thereby helping plants to tolerate the harsh conditions posed by heatwaves. ATT requires a unique set of factors, such as HSFA2, that are not essential for BTT [15–18].

Plants with ATT are considered primed, capable of sustaining the thermotolerant state for several days and exhibit optimized response and resilience when exposed to a subsequent HS [19–21]. The ability to store information of the past stress event during the recovery phase and utilize this information upon a new stress incident, is called memory, and in the case of HS, thermomemory (or HS memory). Thermomemory involves multiple mechanisms, including alterations in chromatin structure, transcriptional and post-transcriptional regulation, as well as processes at the translational, post-translational and metabolic levels [22,23]. The majority of the mechanisms identified so far contribute to somatic memory, which can last from a few days (short memory) to several weeks (long memory), while less is known about the mechanisms that facilitate the transfer of information to the next generation (transgenerational memory) [23].

Here, we explore how thermopriming prepares plants for upcoming HS by improving resilience through optimized thermomemory mechanisms, without compromising growth and development. Progress in physiological, chemical and microbial priming technologies as well as integrating them with genetic methods for plant improvement, like breeding and genome editing, is presented. In combination, these strategies may help to develop climate-resilient crops for future agriculture and global food production under warming temperatures.

## 2. Transcriptional and chromatin-mediated priming and thermomemory

Primed plants may respond to a subsequent stress stimulus with increased speed (faster response kinetics), earlier onset, elevated sensitivity (reacting to lower levels of stress) or stronger response compared to non-primed plants [19,21]. These enhanced responses can involve quicker activation of key genes, elevated phytohormone levels or an increase in defensive metabolites, collectively helping plants adapt to stress more efficiently [22]. Such patterns are typical for responses aimed directly at combating stress, with underlying regulatory networks showing various adjustments, including both up- and down-regulation of factors controlling important gene regulatory networks [24]. Stress memory in plants is manifested through distinct response patterns that are shaped by the priming treatment. Genes involved in transcriptional thermomemory can be divided into two types based on their transcript profiles during priming, the memory phase, and in response to the subsequent severe stress [25–27]. While expression of most HS-induced genes is attenuated within a few hours upon exposure to control temperatures, a subset of genes shows a sustained expression that gradually decreases for a period that can last several days (type I memory). Other genes, characterized as type II, become hyper-activated upon a subsequent stress compared to unprimed plants. Furthermore, another category of memory genes includes those induced in response to a triggering stress treatment only in plants that were previously primed, but not in naive plants [27].

HS causes structural rearrangements in the chromatin that are associated with massive changes in the transcriptome landscape [28]. FORGETTER1 (FGT1) interacts and co-localizes with the chromatin-remodelling complexes BRAHMA (BRM) and ISWI (CHR11 and CHR17) at memory genes including *HSA32* to maintain a nucleosome-free state around the transcription start sites [29,30]. Furthermore, RNA polymerase II (RNAPII)-mediated transcript elongation leads to the recruitment of histone methyltransferases (e.g. ATX1 and ATXR3) resulting in an enrichment of trimethylation of histone H3 at lysine 4 (H3K4me3). H3K27me3 is negatively associated with HS-induced gene transcription and several Jumonji C domain-containing proteins (JMJs) have been identified to regulate H3K27 demethylation [30–34].

Transcriptional memory is associated with histone hypermethylation resulting in H3K4me2 and H3K4me3 marks on memory genes for several days following priming [20,35]. These modifications enable memory genes to stay in an active state, allowing plants to respond more rapidly to an upcoming stress. H3K4me3 is important for enabling the re-induction of transcription by RNAPII [30,31,36,37]. Notably, not all heat-inducible genes exhibit this hypermethylation, for example, the heat-inducible *HSP70* locus in *Arabidopsis thaliana* does not retain H3K4 hypermethylation, suggesting that such modifications are specific to memory genes rather than a general effect of heat-induced transcription [20]. A reduction in H3K4me3 levels at the locus of the *Arabidopsis* memory gene *ASCORBATE PEROXIDASE 2* (*APX2*) results in reduced thermomemory suggesting that chromatin modifications are indispensable for thermomemory [38]. Interestingly, within the same family of genes, such as those encoding small HSPs (sHSPs), some, but not all, are memory genes, suggesting that developing memory is a highly selective and finely tuned process [26].

Among HSFs, *HSFA2* and *HSFA3* (also known as *FORGETTER3*) are indispensable for thermomemory establishment [20,35,39]. Complexes containing both HSFA2 and HSFA3, but also other HSFs, exhibit a more pronounced effect in sustaining the thermopriming response and enhancing thermomemory [35]. HSFA2/A3 complexes recruit a mediator kinase module to the promoters of thermomemory genes by interaction with the CYCLIN-DEPENDENT KINASE 8 (CDK8) subunit and MEDIATOR 12 (MED12) [37]. The mediator kinase module interacts with the pre-initiation complex (PIC) and RNAPII via the core of the Mediator complex (cMED) to initiate transcription, while CDK8 promotes hyper-methylation of H3K4 around the transcriptional start site [37]. The recruitment of the mediator kinase module not only induces chromatin modifications but also promotes RNAPII efficiency which contributes to the hyperactivation of memory genes in case of a new HS incident.

In contrast to the advancements in understanding chromatin-based thermomemory establishment via histone modifications, less is known about other epigenetic mechanisms that may also support the transgenerational inheritance of primed traits [40,41]. Several studies have shown that changes in DNA methylation are related to thermotolerance. In *Arabidopsi*s, heat-induced changes in DNA methylation have been observed in specific genes containing transposon insertions in their promoters. The transposon ONSEN is activated by CHROMOMETHYLASE3 (CMT3) which prevents the methylation of CHH by CMT2 and H3K9me2 accumulation at *ONSEN* chromatin [42–44]. Mutation in the chromatin remodelling gene *DECREASE IN DNA METHYLATION 1b* (*DDM1b*) in tomato enhances thermotolerance of plants exposed to mild chronic HS [45]. However, the specific role of DNA methylation in relation to plant thermomemory remains unknown.

## 3. Post-transcriptional control of thermopriming

While our knowledge of the mechanisms that regulate thermopriming and thermomemory on the transcriptional level is advancing, less is known about other temperature-sensitive processes such as alternative splicing. Priming of *Arabidopsis* plants results in de-repression of splicing after exposure to a new HS [46]. Recently, two serine/arginine-rich splicing factors were shown to regulate the splicing of *HSFA2* transcripts and ATT in tomato [47], suggesting their involvement in priming and memory at the RNA splicing level, though this needs further analyses. Considering that pre-mRNA splicing is mainly co-transcriptional and is influenced by chromatin, it is likely that splicing factors in conjunction with transcription factors, histone modifiers, histone readers, RNAPII and related proteins are involved in memory-related gene expression [48,49].

Another post-transcriptional process controlling thermopriming involves *miRNA156*, which, in association with Argonaute 1 (AGO1), suppresses *SQUAMOSA-PROMOTER BINDING-LIKE 2* (*SPL2*) and *SPL11*, thereby reducing their negative impact on thermomemory gene expression [25]. Additionally, *Arabidopsis* 5'−3' EXORIBONUCLEASE 4 (AtXRN4) facilitates the recovery of *HSFA2* and *HSP70* transcripts to pre-stress levels after thermopriming, which results in reduced heat tolerance [50].

## 4. Metabolic pathways involved in thermopriming

Plant growth and stress responses are energy-intensive processes. Environmental stresses, such as heat, lead to reduced plant growth and reallocation of metabolic resources towards defence- and survival-related processes. Thermopriming modifies this response by establishing a new state of homeostasis, enabling plants to respond more effectively to subsequent HS and recover growth more efficiently. A study by Serrano *et al*. [51] identified significant metabolic shifts in *Arabidopsis* seedlings in response to thermopriming. Primed seedlings exhibited a greater change in metabolites than non-primed plants, both during recovery from thermopriming and upon subsequent heat exposure. Among the metabolites responsive to thermopriming, notable increases were observed in the levels of carbohydrates such as sucrose and raffinose family oligosaccharides (RFOs), and galactinol, a sugar alcohol. Sucrose is an energy source and a carbon skeleton for multiple biosynthetic pathways. Carbon limitation during thermopriming has been associated with growth inhibition. Furthermore, sucrose enhances the effect of thermopriming on the expression of *HSP* genes in the shoot apical meristem (SAM) [52]. The rise in galactinol and RFO levels correlates with an elevated expression of *GALACTINOL SYNTHASE 1* (*GolS1*) following thermopriming, exhibiting a memory pattern that persists for up to 52 h during the recovery phase [35]. This gene is directly regulated by HSFA2 and HSFA3, and its enhanced expression post-priming is diminished in the *hsfa2hsfa3* mutant. GolS is a key enzyme at the start of RFO synthesis, catalysing the conversion of UDP-galactose and *myo*-inositol into galactinol, which then donates galactosyl residues to form RFOs. *GolS1* knockout mutants in *Arabidopsis* show a complete lack of heat-inducible galactinol and raffinose levels. Previous research has linked increased biosynthesis of galactinol and other RFOs to enhanced resistance against oxidative damage under various stresses [53–55]; however, its specific impact on thermopriming-induced HS remains to be further explored.

In wheat plants subjected to thermopriming, an increased activity of sucrose-phosphate synthase and higher sucrose concentrations in flag leaves were observed during post-anthesis HS [56]. Additionally, thermopriming enhances carbohydrate reserve remobilization from wheat stems to grains, leading to greater grain starch accumulation and grain yield [57].

HS impairs photosynthetic activity and causes an overaccumulation of ROS and subsequently oxidative damage [58]. When exposed to HS, primed plants retain increased levels of antioxidant compounds such as glutathione, ascorbate and tocopherols, unlike non-primed plants, which show low levels of these antioxidants [51]. This indicates that priming enhances the plant’s ability to sustain its protective defences against heat-induced oxidative damage. The maintenance of antioxidant activity in primed plants may be linked to the expression of *APX2* [20]. Additionally, research on winter wheat indicates that thermopriming prior to anthesis substantially improves photosynthetic efficiency and antioxidant activities and diminishes oxidative stress in flag leaves during post-anthesis HS [56,59]. Increased levels of other metabolites, such as lipids with glycerol backbones, tricarboxylic acid (TCA) cycle intermediates, tocopherols, flavonoids and phenylpropanoids have also been linked to thermopriming-induced memory and tolerance to HS [51]. Collectively, these findings highlight thermopriming’s significant effects on crucial biological functions, including energy distribution, membrane stabilization and antioxidant activities. Despite these insights, further research is needed to comprehensively identify and characterize thermopriming-associated metabolites across various crops and developmental stages, including tissue-specific responses. These metabolites could serve as molecular markers for breeding plants with enhanced thermotolerance.

## 5. Mechanisms involved in thermopriming at tissue and organ levels

To develop stress-resilient crops, it is essential to understand tissue-specific stress responses and their regulatory interactions. Recent studies have highlighted organ-specific mechanisms and responses during thermopriming.

### (a) Thermopriming in roots

Plant roots play a critical role in water and nutrient uptake, but changes in soil temperatures can alter this process, limiting crop growth. Therefore, the ability of roots to cope with HS is crucial for maintaining plant growth and ensuring efficient nutrient acquisition. Despite this importance, root responses to HS have received comparatively less attention than those of aboveground plant parts.

Recent studies have demonstrated that different species, particularly within the Brassicaceae family, exhibit distinct root responses to HS following thermopriming. For example, in rapeseed (*Brassica napus*), HS leads to increased metabolite exudation, which may help in nutrient acquisition and interaction with soil microorganisms. However, this response did not result in significant changes to root morphology. In contrast, camelina (*Camelina sativa*) exhibited a more conservative root response, with notable changes in organic acid exudation, indicating a strategy aimed at enhancing stress tolerance [60]. In maize, another key agricultural crop, thermopriming through mild heat exposure resulted in significant improvements in root morphology, including increased total root length and enhanced root distribution in deeper soil layers. These changes are crucial for improving the plant’s ability to access water and nutrients from deeper soil horizons, thus strengthening its overall functionality under HS conditions [61]. In addition to these studies, research on the vegetable crops *Lactuca sativa* (lettuce) and *Eruca sativa* (arugula) further supports the efficacy of root-zone-specific HS priming. Root-zone priming of these plants enhanced productivity and photosynthetic performance under hardening treatment at 42°C [62]. This suggests that targeted HS priming strategies, especially at the root level, could be used to enhance crop productivity in the face of hardening temperatures.

The above findings collectively highlight that while HS is often believed to be primarily sensed and responded to by aboveground plant tissues, it is becoming increasingly clear that root functions are also significantly impacted. Therefore, the ability of roots to adapt to HS through changes in morphology, exudation and signalling is crucial for maintaining plant health and productivity. Understanding root-level responses will be essential for developing heat-tolerant crops.

### (b) Shoot apical meristem

The SAM, a pool of undifferentiated cells at the shoot tip, is crucial for plant growth and recovery after stress. Recent reports show that the SAM adapts its molecular responses to thermopriming [52,63]. Transcriptome analysis by Olas *et al*. [52] revealed that the SAM of *Arabidopsis* responds faster and, to some extent, differently to thermopriming than whole seedlings, enabling quicker recovery from subsequent HS. This recovery process is supported by thermopriming-induced restoration of stem cell regulators, such as *CLAVATA1* (*CLV1*) and *CLV3,* which are typically downregulated after HS. The reactivation of these regulators is essential for maintaining stem cell activity and promoting regrowth. Among the genes induced by thermopriming in the SAM is *FRUCTOSE-BISPHOSPHATE ALDOLASE 6* (*FBA6*), a key player in carbohydrate metabolism [52]. The critical role of carbohydrate metabolism and sugar availability for sustaining thermopriming and establishing thermomemory was validated in *fba6* mutants grown in the presence of carbon, and in wild-type plants grown under sugar-deficient conditions, i.e. lack of sucrose or removal of cotyledons, both of which lead to weakened seedling survival and delayed recovery of leaf formation following HS in thermoprimed plants. Notably, HSFA2 directly regulates *FBA6* expression within the SAM, highlighting a novel tissue-specific regulatory module crucial for the HS response [52].

Furthermore, thermopriming activates HSFA7b in the SAM, enhancing ethylene signalling by upregulating *ETHYLENE-INSENSITIVE 3* (*EIN3*) expression and ensuring a balanced response through the induction of negative regulators like *ETO1* and *EOL1* [63]. Exploring the interplay between HSFA2-mediated control of carbohydrate metabolism and HSFA7b-driven ethylene signalling may reveal important insights into how metabolic and hormonal pathways integrate to support thermopriming at the SAM. Additionally, other HSFs, including *HSFA1e*, *HSFA3*, *HSFA7a*, *HSFB1*, *HSFB2a* and *HSFB2b* are induced by thermopriming at the SAM [52], highlighting the complexity in these signalling networks.

### (c) Reproductive tissues

HS adversely impacts both male and female gametophytes, significantly reducing yield potential [64–68]. Despite the critical importance of reproductive tissues for yield formation, only a limited number of reports have described the effects of thermopriming on reproductive development, and the genetic and molecular mechanisms underlying the impact of thermopriming on reproductive tissues remain largely unknown. In wheat, thermopriming at the early reproductive stage enhances thermotolerance at post-anthesis and prevents seed losses [56]. Additionally, research in tobacco demonstrated a positive impact of thermopriming on pollen functionality when exposed to heat. HS disrupts pollen metabolism by lowering sugar levels, particularly sucrose, and reducing calcium (Ca²^+^) concentration and distribution, impairing actin filament dynamics and leading to slowed pollen tube growth. However, thermopriming mitigates these effects by rebalancing Ca²^+^ and ROS levels [69].

The impact of thermopriming at the early stages of pollen development remains largely uninvestigated. Pollen is very sensitive to high temperatures, particularly during the early stages of development (microgametogenesis) as well as during germination. Interestingly, several HS-responsive mechanisms are active in the absence of stress [17,70,71]. HSFs activate the expression of *HSPs* and other HS-induced genes during meiosis and while transcription ceases after the tetrad stage, several HSF and HSP proteins can be detected in mature pollen [17,72]. Currently, the developmental regulation of HSF-HSP networks during microgametogenesis is considered as a priming mechanism that enhances the thermotolerance capacity of the male gametophyte during an upcoming HS.

Phytohormones play a critical role in priming reproductive tissues, particularly pollen, for HS, offering a promising strategy to enhance crop resilience to high temperatures. Exogenous application of abscisic acid (ABA) in rice spikelets mediates sugar metabolism due to regulation of cell wall invertases, sucrose synthases and sugar transorters, thereby reducing pollen abortion under HS [73]. Exposure to HS results in a reduction of expression of genes involved in auxin biosynthesis in developing anthers of barley and *Arabidopsis* and consequently the levels of endogenous auxin levels are also reduced [74]. The application of exogenous auxins prior to HS enhances thermotolerance [74]. Ethylene is essential for pollen acclimation. In tomato, ethylene-insensitive mutants exhibit reduced pollen thermotolerance due to sucrose depletion, while exogenous ethylene enhances resilience as shown in tomato [75]. Gibberellins (GAs) and jasmonates (JAs) further mediate pollen HS responses. GA deficiency mimics HS effects, disrupting tapetal function and reducing pollen viability, while mild HS represses GA-responsive tapetum genes and B-class MADS-box genes, key regulators of pollen development [76,77]. JA enhances pollen antioxidant capacity, reducing HS-induced oxidative damage [78,79]. Altogether, hormone-driven priming mechanisms provide a framework to enhance reproductive success and yields under increased temperature.

Although direct evidence is currently missing, it can be hypothesized that pollen may be epigenetically programmed to retain a memory of past stress to better adapt to recurring or novel stress waves. Alongside *HSFs* and *HSPs*, genes involved in the unfolded protein response (UPR), ROS scavenging and pathways for the synthesis of secondary metabolites, such as flavonoids, auxin and abscisic acid, are upregulated by default as pollen matures. These processes are likely triggered by Ca^2+^-dependent calmodulin (CaM3), Ca^2+^-dependent protein kinases (CDPKs) and H_2_O_2_-induced mitogen-activated protein kinases (MAPKs) [80,81]. Nevertheless, this priming mechanism alone is not sufficient to provide adequate protection, particularly in grain crops, where thermotolerant pollen is required to ensure high yields [71].

## 6. Role of cellular signalling and memory in priming

Ca^2+^ ions play important roles as second messengers in plants, regulating many physiological processes [82,83]. HS triggers an elevation of Ca^2+^ levels in the chloroplast stroma, and this increase involves the calcium-sensing receptor protein CAS; the calcium response to heat is reduced in *cas* mutants of *Arabidopsis* [84]. A recent study found that the *cas* mutant produces higher biomass than wild-type plants after thermopriming, and that it accumulates higher levels of HSP17.6, a marker for the response to HS. This observation reveals a role of CAS in thermomemory [85]. Furthermore, levels of free amino acids did not increase in *cas* mutants, in contrast to wild-type plants, indicating a lower autophagic activity. Autophagy has previously been identified as a key player in the recovery of plants from HS [86,87], and an inhibition of autophagic activity in the *cas* mutant, which leads to increased HSP17.6 level, can explain their increased thermopriming capacity.

Another study suggested the involvement of Ca^2+^ signalling in the response of rice pollen to HS [88]. Knocking out *RICE MYO-INOSITOL-3-PHOSPHATE SYNTHASE 2* (*RINO2*) exacerbated the negative effects of HS on pollen germination and tube growth. This was accompanied by reduced levels of phosphatidylinositol 4,5-bisphosphate (PI (4,5) P_2_), disrupting the typical Ca^2+^ gradient at the apical region of pollen tubes and the arrangement of actin filaments necessary for efficient pollen tube growth [88]. RINO2, therefore, appears to play a critical role in supplying phosphatidylinositol (PI) derivatives essential for the survival and proper functioning of pollen under HS. Investigating whether RINO2 is also required for thermomemory in pollen will be of great interest in the future.

In addition, multiple reactive molecular species, including ROS (e.g. hydrogen peroxide), reactive nitrogen (RNS, e.g. nitric oxide) and sulfur species (RSS, e.g. hydrogen sulfide) and reactive carbonyl species (RCS, e.g. methylglyoxal) represent important signalling molecules in abiotic stress responses including priming during HS and other abiotic stresses [80,89–92]. A recent study by Bi *et al*. [93] observed a role of FaHSP17.8-CII, a small HSP in the cool-season grass tall fescue (*Festuca arundinacea*), in the memory of priming-induced ROS accumulation and photosystem II (PSII) electron transport. Of note, *FaHSP17.8-CII* is a transcriptional thermomemory gene. During heat priming, elevated levels of H3K4me3 at the start of the gene’s coding region occur. Knocking out *FaHSP17.8-CII* enhances chloroplast damage under HS compared to wild type, whereas overexpression of *FaHSP17.8-CII* mitigated the damage. The research also revealed that FaHSP17.8-CII affects the expression of genes involved in ROS signalling in chloroplasts during thermomemory. Thus, the transcriptional memory of *FaHSP17.8-CII* appears to play a crucial role in safeguarding chloroplasts from HS-induced damage [93].

Glucose is another signalling molecule during thermomemory, acting via a TARGET OF RAPAMYCIN (TOR)—E2Fa transcription factor module [94]. After thermopriming, glucose-activated TOR activates E2Fa by phosphorylation, enhancing the expression of downstream genes like *HSFA1s* and *HSFA2*. This elevates *HLP1* expression, which supports thermomemory by promoting lysine acetylation on histone H3 (H3K9, H3K14, H3K18, H3K23, H3K27) at HS gene promoters, activating their transcription. HLP1 also maintains H3K4me3 marks at thermomemory genes, sustaining their expression [44].

Phytohormones play crucial roles in developmental programmes and stress responses [95–100]. An example is ethylene, which is important for thermomemory at the SAM [63]. BRI1 EMS-SUPPRESSOR 1 (BES1), a key regulator in brassinosteroid signalling, maintains memory gene expression by demethylating histone H3K27me3 [101]. BES1 can also be activated independently of brassinosteroids through ABA-repressed phosphatases [102] and, once active, binds HSFA1s to induce HS proteins like HSP70 and HSP90.

A rapid activation of *HSFA2* in the *Arabidopsis* shoot apex, followed by delayed activation in other tissues, suggests the presence of a mobile signal moving from the apex to other organs to induce *HSFA2* [52,103]. Nitric oxide (NO) or its cellular reservoir, S-nitrosoglutathione (GSNO), act as signalling molecules, with NO levels rising after HS; NO-deficient mutants show reduced *HSFA2* expression. The transcription factor GT-1 interacts with the *HSFA2* promoter in an HS-dependent manner. S-nitrosylation of GT-1 by GSNO enhances its binding to NO-responsive elements indicating a redox-based signalling mechanism in the HS response [103].

## 7. Trans-priming thermotolerance

In nature, HS often occurs sequentially, or simultaneously, with other stresses or environmental stimuli. Therefore, it is not surprising that core elements of drought and HS response pathways are, in part, common or overlapping [104]. For example, *Arabidopsis DREB2A* is both a drought- and HS-responsive gene, which among others regulates *HSFA3*, while bZIP transcription factors controlling UPR are activated by heat and other abiotic stresses that cause proteotoxicity in the endoplasmic reticulum (ER) [11,105–109]. In barley, HvHSFA2e regulates heat and drought tolerance by modulating phytohormone and secondary metabolic pathways [110]. Thus, thermotolerance can be primed by exposure to other stresses, a phenomenon called trans- or cross-priming. In wheat, plants at the stem elongation stage exposed to drought are primed for thermotolerance at the grain filling stage to support increased yields in comparison to non-primed plants [111,112]. Interestingly, thermotolerance can also be primed by exposure to low temperatures, either by regulating the synthesis of proline, salicylic acid (SA), phospholipase D, HSPs, or by increasing activities of oxidative damage-preventing enzymes as shown for tomato and grape [91,113].

While trans-priming relies on a stress stimulus different from the one triggering stress memory, environmental inputs that do not induce stress can also prime plants for thermotolerance. Plants constantly sense and adapt to both anticipated and unexpected daily and seasonal fluctuations in light and temperature, adjusting their morphology and development accordingly.

Light quality, intensity and photoperiod are major factors affecting plant productivity, fitness and nutritional quality. Plant responses to light and high temperatures are interlinked at many levels including perception, signalling and in some cases memory [114,115]. Key light signalling components including the red/far-red light receptor and thermosensor phytochrome B (PHYB), major growth regulating transcription factors (PHYTOCHROME INTERACTING FACTORs, PIFs), the EARLY FLOWERING 3 (ELF3) clock component and chromatin regulators in *Arabidopsis*, which are conserved in angiosperms, play major roles in high-temperature responses [116–118]. In addition to their role in the transcriptional regulation of gene expression, PHYB and PIFs control chromatin accessibility, condensation and gene positioning in response to light and high temperatures [119–121]. More specifically, red light signalling factors modulate the removal of the repressive histone variant H2A.Z from the nucleosomes of HS-responsive genes, leading to their induction [121,122]. Furthermore, the master regulators of heat shock responses, HSFA1s, can promote thermomorphogenesis by directly interacting with PIF4 [116]. In addition to the established role of PHYB in thermosensing and signalling, the blue light cryptochrome receptors were recently shown to control thermotolerance by mediating the nuclear import of HSFA1d in a heat-triggered and blue light-specific manner [123]. Lastly, the JMJ18 H3K36me2/3 demethylase known to regulate light- and temperature-controlled flowering initiation has been shown to promote heat resilience in *Brassica rapa* without compromising plant growth [118].

These above examples illustrate the interdependence of light and high-temperature signalling and highlight their potential as targets for enhancing plant resilience to extreme environmental conditions related to HS. Beyond genetic engineering approaches to improve crop resilience to high temperatures, leveraging the interconnected light and temperature signalling pathways offers a novel, non-invasive alternative.

Light plays a role in modulating plant heat responses in a wavelength-dependent manner. More specifically, the red light/thermo-sensor PHYB negatively regulates responses to high temperatures, whereas blue light promotes thermotolerance through the action of CRYPTOCHROME 1 (CRY1) [11,116,119]. Designing a bespoke wavelength-customizable natural approach to deliver a light-tunable system that modulates the duration and magnitude of HS responses in plants without impairing growth and development is a feasible eco-friendly option that could be a future strategy for applications in (semi-)controlled environments. However, to achieve this and to optimize and effectively implement this light-tunable system, further research is essential to elucidate the exact molecular and physiological events underpinning light-controlled HS responses and thermomemory in *Arabidopsis*. Expanding this knowledge to crop-relevant species will be important for future applications in agriculture.

## 8. Chemical and microbial priming for thermotolerance

Chemical and microbial priming agents improve the thermotolerance capacity of plants by activating a variety of molecular and physiological mechanisms related to HS resilience [124]. Low concentrations of reactive oxygen, nitrogen and sulfide species (RONSS), such as H_2_O_2_, nitric oxide (NO), and hydrogen sulfide (H_2_S) serve as signalling molecules that activate HSFs and in turn their corresponding regulatory networks including many HSPs [125], with an example being the study of Christou *et al*. [126], whereby strawberry root pre-treatment with the H_2_S donor NaHS resulted in increased thermotolerance and transcriptional regulation of *HSPs*, genes coding for proteins with antioxidant activity and aquaporins. Phytohormones, such as SA, brassinosteroids and GAs, also regulate stress-responsive gene expression [97]. SA, for instance, activates transcription factors that enhance the antioxidant capacity of plants, reducing oxidative damage by increasing enzymes like superoxide dismutase (SOD) and catalase (CAT), which neutralize ROS generated by HS [127]. Seed priming in combination with ascorbic acid and SA treatments improves rice growth under HS [128]. In addition to phytohormones, other treatments have been found to enhance plant thermotolerance. For example, *Arabidopsis* plants grown *in vitro* and pre-treated with ethanol showed enhanced HS tolerance by activating UPR signalling via putrescine accumulation, leading to enhanced HS tolerance [129]. Furthermore, exogenously applied volatile organic compounds (VOCs) like (E)-2-hexenal activate transcription factors like *HSFA2*, which upregulate stress-response genes, thereby preconditioning plants to robustly respond to HS [130]. Additionally, specialized metabolites like flavonols, applied exogenously or upregulated within plants, help them to withstand HS. In tomato, overexpression of *FLAVANONE 3-HYDROXYLASE* (*F3H*) increases endogenous flavonol production, protecting pollen from heat-induced impairments in germination and tube elongation. Flavonol-mediated protection from HS in tomato functions specifically within pollen grains with all three flavonols (kaempferol, quercetin and myricetin) protecting mature pollen from high-temperature stress [131]. Exogenous application of flavonols to tomato pollen complements flavonol production in *anthocyanin reduced* (*are)*, the mutants with impaired endogenous flavonol synthesis, and confers thermotolerance by improving pollen germination and pollen tube length [131]. This protection is achieved by reducing excess ROS and maintaining ROS homeostasis, essential for cellular stability during HS. Additionally, flavonols also trigger a robust transcriptional response, modulating the expression of key heat-responsive genes, including *HSP101*, *HSP70* and *APX1*, which contribute to protein stabilization and antioxidant defence, potentially priming plants for improved heat tolerance [131].

Beneficial microbes, including plant growth-promoting rhizobacteria (PGPR) and arbuscular mycorrhizal fungi (AMF), can promote thermotolerance through biochemical interactions with host plants [132–134]. Microbial inoculation promotes thermoregulation by activating stress responses, enhancing nutrient uptake and modulating antioxidant activity, which together reduce the impact of HS. In wheat, a consortium of different AMF, including *Rhizophagus irregularis*, *Funneliformis mosseae*, *F. geosporum* and *Claroideoglomus claroideum*, alleviates HS-induced damage, improves plant source-sink relationships and increases grain number [132]. Positive effects on thermotolerance have been reported for the rhizobacterium *Bacillus cereus* SA1 in soybean and *Pseudomonas putida* AKMP7 in wheat. Inoculation of *Arabidopsis* and wheat with the endophyte *Enterobacter* SA187 increased plant biomass, height and grain yield under HS [135–137]. This enhancement in thermotolerance is attributed to transcriptome changes that are associated with H3K4me3 levels at thermomemory loci through an HSFA2-dependent pathway [137]. The thermotolerance induced by SA187 relies on ethylene signalling via the transcription factor ETHYLENE INSENSITIVE 3 (EIN3), which results in stable H3K4me3 modification at thermomemory genes, stimulating a lasting priming effect. Importantly, plant growth-promoting bacteria that contain 1-aminocyclopropane-1-carboxylate (ACC) deaminase can lower the ethylene levels of plants by degrading ACC, the precursor of ethylene [138]. In addition, some bacteria produce and secrete the auxin indole-3-acetic acid (IAA), which in turn promotes growth and contributes to abiotic stress resilience.

## 9. A perspective lab-to-farm strategy towards primed thermotolerant crops

Priming is a promising approach for enhancing resilience to HS by preconditioning plants to respond more rapidly and effectively to future high-temperature events [23]. While the concept is not new, recent advancements in our understanding of the molecular mechanisms, technological innovations in delivering priming agents with high efficacy, and the discovery of beneficial microbes that stimulate thermotolerance have opened a new era in crop priming. The new findings offer a realistic toolbox for farmers to mitigate the negative effects of climate change on crop production. In this context, we propose a roadmap for leveraging priming approaches in agriculture towards climate-proof crops, with a focus on HS.

### (a) Traits related to thermopriming capacity

Priming for thermotolerance has been associated with physiological and biochemical traits such as improved membrane stability, increased accumulation of HSPs, efficient ROS scavenging and increased antioxidant enzyme activity [69]. Such traits can serve as universal breeding targets across multiple crops as they help plants to maintain PSII stability, as well as a more efficient stomatal conductance and carbon fixation during an upcoming heat incident. However, it is important to identify specific traits for priming, depending on the particular crop species and varieties, and the part of the plant that contributes to yield (e.g. roots, grains, fruits, leaves). For instance, priming strategies that enhance pollen viability, fertilization efficiency or synchronize flowering with favourable conditions can mitigate yield losses in cereal crops like wheat and rice [139]. For fruit crops such as tomatoes and grapes, enhanced fruit set, reduced flower drop and physiological disorders such as blossom-end rot are important priming traits against high temperatures. Priming can enhance root architecture by promoting increased branching and elongation, thereby improving the plant's capacity for efficient water uptake during hot periods which may be accompanied by water shortage. Priming can also adjust developmental timing such as flowering time and grain filling, which can help farmers optimize planting schedules or implement targeted interventions to align critical growth stages with favourable environmental conditions [140,141]. This adaptive approach is particularly important given the increasing frequency and severity of heatwaves, as well as unpredictable fluctuations in weather patterns caused by climate change. By integrating priming strategies, farmers can make year-to-year adjustments to account for these variations, ensuring that crops can withstand extreme conditions and maintain yield stability, even in the face of rapidly changing climates. For example, priming may selectively simulate the adaptive traits of early flowering barley varieties that escape late-season heatwaves, or late-flowering millet varieties that avoid HS during seed setting.

### (b) Developing priming protocols for crops

Establishing effective priming protocols for enhancing thermotolerance requires systematic and crop-specific approaches, integrating physiological, biochemical and molecular analyses. Priming strategies should target optimized thermotolerance while minimizing trade-offs related to growth, yield and quality of the produce. This is particularly important when cross-priming by other abiotic stresses such as drought or salinity is considered. Furthermore, while a mild heat exposure is very efficient as it maximizes the activation of relevant HS tolerance mechanisms under laboratory conditions, it can be costly or even unsuitable under farm or greenhouse conditions. Therefore, optimizing priming via genetic means, e.g. by utilizing varieties with increased priming capacity, combined with chemical and microbial interventions, might be the most feasible and scalable approach for various agricultural settings in the future.

Technological tools like biosensors, remote sensing and machine learning models can facilitate real-time monitoring of plant health and aid farmers in making informed decisions [142]. Understanding the thresholds for priming-induced stress memory, as well as its duration, is critical for designing intervention programmes that align with crop growth stages and can fully harness the priming capacity of a crop. Precision agriculture tools, such as environmental sensors and remote monitoring systems, can assist farmers in identifying the most suitable timing and dosage of priming treatments in real time, thereby maximizing their efficiency. Field trials across diverse agro-ecological zones can further validate protocols and ensure their adaptability and scalability for different farming systems.

**Figure 1.**
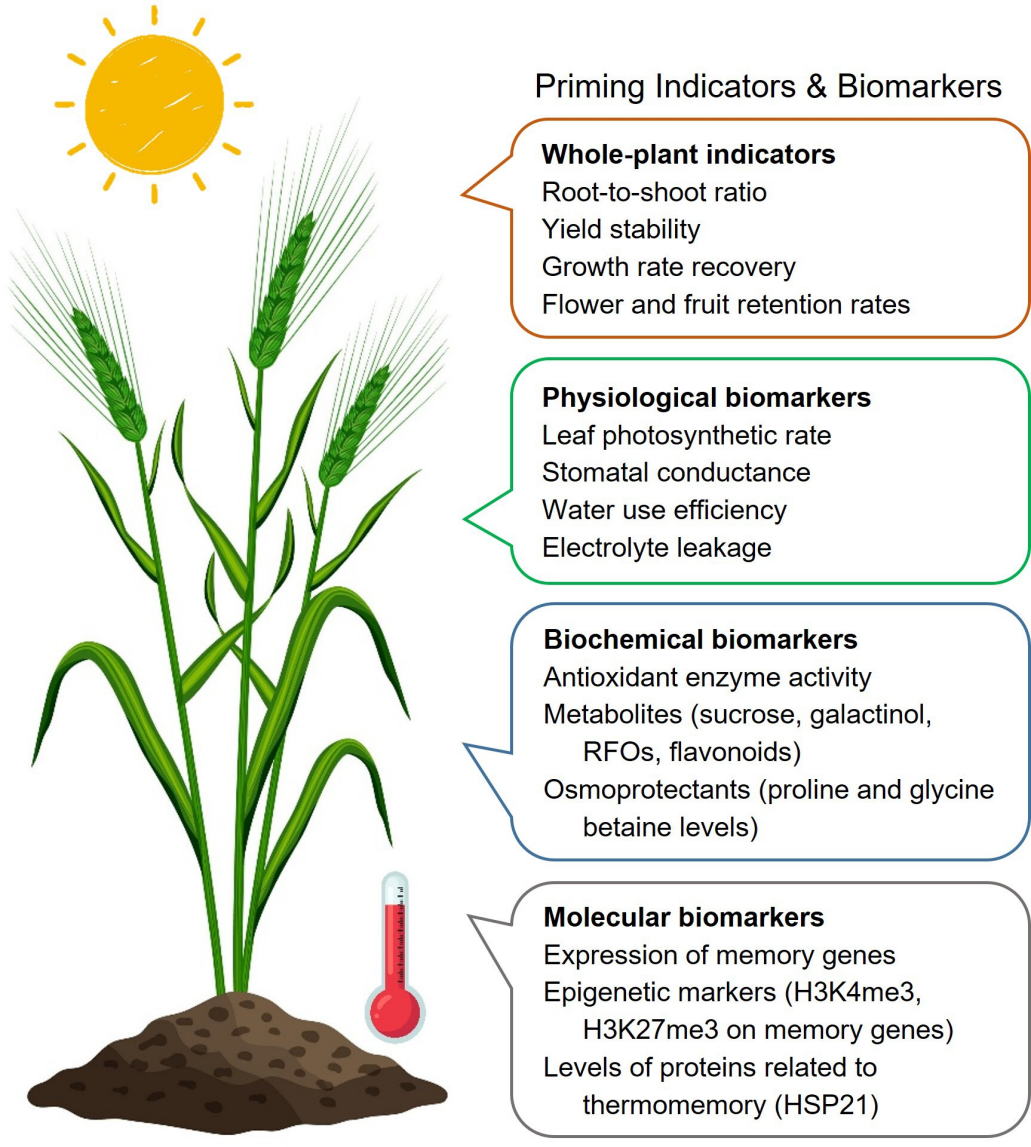
Phenotypic indicators and biomarkers of priming status and thermomemory of plants. Vector images were designed by Freepik (free licence; www.freepik.com).

Integrating single-cell analysis into priming research can provide valuable insights into the most thermosensitive tissues and cell types, such as pollen, ovules or stem cells [143]. This approach can identify cell populations with enhanced priming capacity and their role in propagating the primed state to daughter cells or neighbouring tissues. By targeting these specific cells with genetic, physiological or chemical interventions, researchers can design more precise and effective priming strategies, ultimately contributing to the development of climate-resilient crops that maintain productivity and quality under HS conditions. Establishing a set of universal and identifying species-specific indicators and biomarkers will be crucial for monitoring the priming status of the plant and thermotolerance (figure 1).

### (c) Breeding for priming

Currently, the majority of genetic models for priming are based on the non-crop model plant *A. thaliana*. Considering that the main features of the HS response and thermotolerance are conserved among plant species, such as HSFA1’s role as a master regulator [12,13,144,145], and HSFA2’s function in ATT [17,39], it is very likely that the chromatin and transcriptional memory models apply also to crops. This indicates that priming strategies developed in model plants can be adapted and refined for use in crops. Integrating priming concepts into breeding programmes represents a promising avenue for enhancing crop thermotolerance in the future. Screening for natural and induced variation in priming capacity across different plant genotypes will allow breeders to select for traits that enhance thermomemory and resilience in agricultural settings. Advanced genomic tools, such as genome-wide association studies (GWAS) and CRISPR-Cas-based editing can be utilized to identify and modify key regulatory genes involved in priming mechanisms. In tomato, natural variation between wild and semi-domesticated species, and modern cultivars in ATT has been attributed to HSFA2 haplotypes [146], while HSP101 and HSA32 are associated with variations in long-term ATT in rice varieties [147]. These studies support a breeding-based approach for enhancing the priming capacity and thermotolerance of crops.

Gene editing approaches such as CRISPR can provide a more direct means to manipulate gene expression or protein activity. Enhancing or reducing central regulators of memory and adjusting the activity of priming-related regulatory gene networks will allow the extension of the memory period. Identifying how plants balance sustaining memory elements, such as H3K4me3, against resetting mechanisms like H3K27me3, will allow the fine-tuning of thermotolerance without compromising growth and productivity. Additionally, exploring how DNA methylation patterns contribute to resetting and sustaining stress memory could lead to targeted interventions that enhance priming capacity while minimizing trade-offs. Insights gained by gene editing can also help breeders to create new breeding pipelines for the development of crop varieties with optimized thermotolerance that are resilient to the increasing frequency and severity of HS incidents.

## 10. Combining genetic and advanced priming strategies to secure crop yields in a hotter world

**Figure 2.**
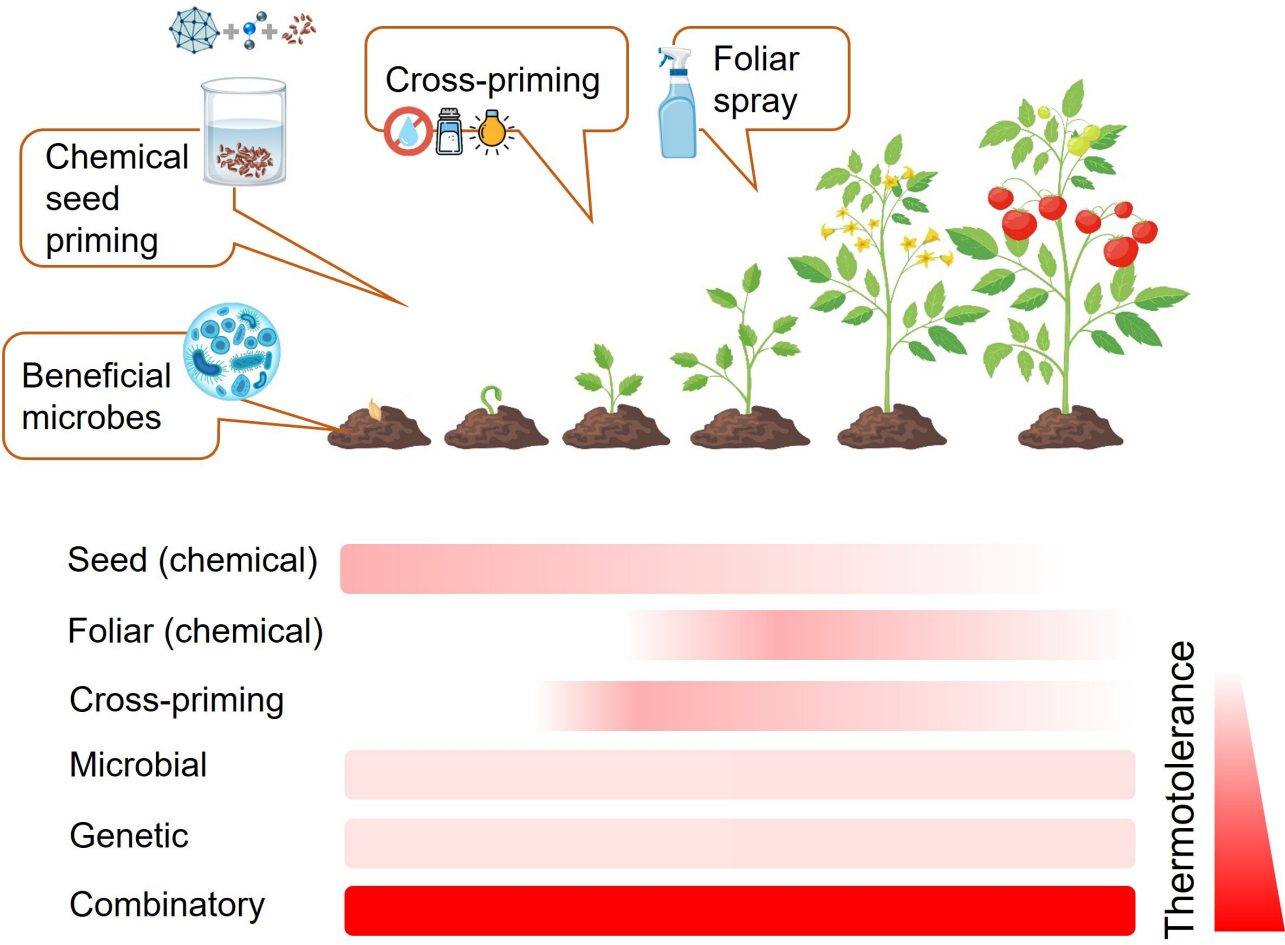
Hypothetical model of the synergistic effects of multiple priming strategies on crop thermotolerance. Thermotolerance capacity is depicted on a white (sensitive) to red (maximum) thermotolerance scale. Vector images were designed by Freepik (free licence; www.freepik.com).

Combining breeding for thermotolerance with advanced priming strategies and technological innovations offers a holistic solution to enhance agricultural resilience against climate change (figure 2). By integrating genetic advancements, such as breeding or editing for thermotolerance traits and improved priming capacity, with optimized application protocols for foliar sprays, microbial inoculants and chemical agents, the preparedness of crops against heatwaves can be dramatically improved (Boxes 1; 2). Machine learning and AI further amplify this approach by enabling real-time monitoring, precise application and pro-active and anticipatory decision-making. These tools ensure that interventions are tailored to specific crop types, growth stages and environmental conditions, maximizing efficiency and ensuring yields. Together, these methods create a synergistic system where genetic enhancements provide a strong baseline for resilience, while adaptive priming techniques and precision agriculture technologies offer dynamic and adaptive responses to fluctuating conditions. We propose that this integrated approach will secure sustainable crop production and food security in the face of global climate challenges.

Box 1 **. Prime-time: when to apply priming agents.**

**Seed stage**: Priming seeds before sowing supports early germination, improves seedling vigour and favours uniform seedling establishment. Techniques like hydropriming, osmopriming and treatment with phytohormones (e.g. SA, GAs) or antioxidants (e.g. ascorbic acid) are effective particularly when combined with state-of-the-art nanocarriers for achieving optimum efficacy [148].

**Seedling establishment stage**: During early vegetative growth, priming agents can support the development of robust root systems and stress-adaptive architecture, crucial for water and nutrient uptake during hotter periods [149]. Microbial inoculants, such as PGPR can boost root health and nutrient uptake, increasing the resilience of the crop in case of an upcoming heatwave [132–134].

**Vegetative stage**: Application of chemical priming agents during the vegetative stage can enhance photosynthetic efficiency, antioxidant capacity and ROS scavenging systems. Foliar sprays with RONSS, phytohormones or specific metabolites like flavonoids can stimulate thermotolerance capacity without trade-offs on growth [89].

**Reproductive stage**: Priming agents can target pollen viability, fertilization efficiency and protection of reproductive structures. Heat-sensitive tissues, such as pollen grains and ovules, can benefit from treatments with calcium, flavonoids or specific microbial formulations that stabilize ROS levels and improve metabolic activity [69,81].

Box 2 **. Strategies and technologies for priming.**

**Seed treatments**: Seed coatings with microbial inoculants, antioxidants or phytohormones deliver priming agents directly to seeds, ensuring uniform and controlled exposure and optimized priming [148,150].

**Foliar sprays**: These are effective for delivering phytohormones, antioxidants or flavonoids directly to leaves during vegetative and reproductive stages [151]. Foliar sprays offer a flexible and rapid method for mitigating the effects of weather fluctuations and allow farmers to make short-term decisions to protect crops. Combined with machine learning models which analyse weather forecasts, sensor data and crop conditions, farmers can be advised for optimal spray timings and formulations [152].

**Soil and root treatments**: Soil enrichment with beneficial microbes such as PGPR, organic acids or nutrient-enriched formulations enhances root health and stress resilience [136]. Drip irrigation systems can deliver soluble priming agents directly to the root zone, reducing water use and ensuring targeted application with maximum efficacy [153]. Machine learning can propose the best combination of microbial inoculants and chemical mixtures based on the crop, soil status, current and forecasted environmental conditions [154].

**Controlled environment applications**: In greenhouses, temperature and humidity control systems can be synchronized with priming treatments to simulate mild HS, enhancing the activation of thermotolerance pathways. Both in the field and in greenhouses, adjustments in the irrigation programme for induction of priming by mild drought and salinity stress can be used for cross-priming [23]. Adjustments in light quality and intensity can stimulate HS responses in plants and prime crops for thermotolerance [123].

## Data Availability

This article has no additional data.
